# Effect of thermal treatment with water, H_2_SO_4_ and NaOH aqueous solution on color, cell wall and chemical structure of poplar wood

**DOI:** 10.1038/s41598-018-36086-9

**Published:** 2018-12-07

**Authors:** Jiangtao Shi, Yu Lu, Yaoli Zhang, Liping Cai, Sheldon Q. Shi

**Affiliations:** 1 College of Materials Science and Engineering, Nanjing Forestry University, Nanjing, 210037 China; 2 Mechanical and Energy Engineering, University of North Texas, Denton, TX 76207 USA

## Abstract

Thermal treatments with water, diluted acid, and diluted alkali aqueous solution of poplar wood blocks were carried out in a Teflon-lined autoclave at three temperatures. The effects of different liquids and temperatures on wood surface color, cell wall microstructure, and chemical structures were investigated by the chromameter, scanning electron microscope (SEM), and Fourier transform infrared spectroscopy (FTIR). From the chromameter, it was observed that the lightness value decreased with temperature for all treatment conditions. The a* value increased with temperature in all liquid treatments. The b* value increased with temperature in hydrothermal and thermal with H_2_SO_4_ treatment but decreased with temperature in thermal with NaOH treatment. The total color difference (Δ*E*) was slightly changed in the hydrothermal treatment, but dramatically changed in the thermal with H_2_SO_4_ and NaOH aqueous treatments. SEM showed that the cell wall structure was damaged differently with different reagents and temperature. Middle lamella layers were always fractured in hydrothermal and NaOH treatments. However, both middle lamella and secondary cell wall were damaged after the H_2_SO_4_ treatment and intensified with temperature. These fractures usually parallel with the S2 layer microfibril angle (MFA) in the fiber cell wall. The FTIR analysis suggested that the chemical structure was obviously changed after the thermal with H_2_SO_4_ and NaOH treatments. And the missing or decreasing C=O absorption peak indicated hemicellulose is degraded and new compounds produced during thermal with H_2_SO_4_ and NaOH treatment. On the other hand, lignin was partly degraded in the H_2_SO_4_ treatment and guaiacyl nuclei was degraded before syringyl nuclei.

## Introduction

As an efficient method for wood modification, hydrothermal technology is employed to deposit various metal oxides on wood surface, aiming at fabricating multi-functional wood, such as dimensional stability, anti-UV, hydrophobic, decomposition of formaldehyde, etc^1–5^. Be different than traditional wood hydrothermal methods, recently technique is sealing wood with water or other chemical solvents into a Teflon-lined autoclave and heating at relatively low temperature from 80 °C to 120 °C^1,2^. One of the important reasons for selecting of this temperature is wood tissue decomposed drastically when over 160 °C hydrothermal treatment^6^. Although the relatively low temperature range was selected during the process of thermal treatment, the autogenous pressure in a small closed chamber make the liquid into the wood cell gaps or through the grooves, which may cause more cracks in the intercellular layer and the pits^7,8^. Combination of moisture with exposure to heat will be impact on the degradation of all components of wood^9,10^.To our knowledge, more attention focus on the synthesis of various ingredients on wood and its function while limited to know the effect of this treatment condition on wood itself.

On the other hand, the wood cell wall structure and wood color will be changed when facing various modification conditions, such as the heat treatment, at the acid or alkali liquid environment, and light or CO_2_ laser irradiation. Wood color is one of the important properties related to the wood utilization and modification. Several studies^11–16^ reported that the cleavage of chemical bonds in polymers causes the changes in chemical structures of lignin and hemicellulose, and the formation of new chromophore substances can be responsible for color changes of wood. In addition, the changes of wood color in hydrothermal at low temperature may be caused by moving out of extractives in wood. Ji *et al*.^17^ investigated dilute acid and dilute alkali pretreatment of *Miscanthus* × *giganteus* resulting in partial degradation of the low degree substitution xylose in the cell wall. The connection bond between lignin and hemicellulose was cleaved. As a result, the cell wall porosity and the permeability increased. Saito *et al*.^7^ suggested that the ultrastructure of the inner surface of the fiber cell walls was changed after the dilute acid, dilute alklic or hydrothermal pretreatment. Ma^18^ found that the fiber cell wall of Triploid *Populus tomentosa* was destroyed after the NaOH thermal treatment, but no effect on cell corner middle lamella (CCML). The chemical pretreatment combined with disc refiner mechanical method was also employed for reducing energy and increasing enzymatic digestibility of wood^19,20^. Although these studies focused on the changes of wood color, chemical constituents and conversion efficiency of woody tissue, the cell wall structure variations in wood block during the thermal with different liquid treatment is still unclear.

Based on the previous reports, it can be assumed that there may be structure damage and composition changes on cell walls during the reaction process of wood thermochemical modification, which probably are closely related with the treatment temperature and solutions. Furthermore, wood color will be changed after facing different thermochemical modifications. As an explorative experiment, we selected the concentration and temperature of acid and alkali treatment refer to some wood degradation publishes. Herein, the objectives of this study were: 1) to observe and quantify the changes of color on wood surface and microstructure of cell walls after the thermal treatment with water, dilute acid and alkali; and 2) to describe the changes of chemical structure on wood surface after those pretreatments. The results of this work not only can provide theoretical foundation in wood thermochemical modification but also can be used as a method for the wood surface coloring.

## Results and Discussion

### The wood color changes

**Figure 1:**
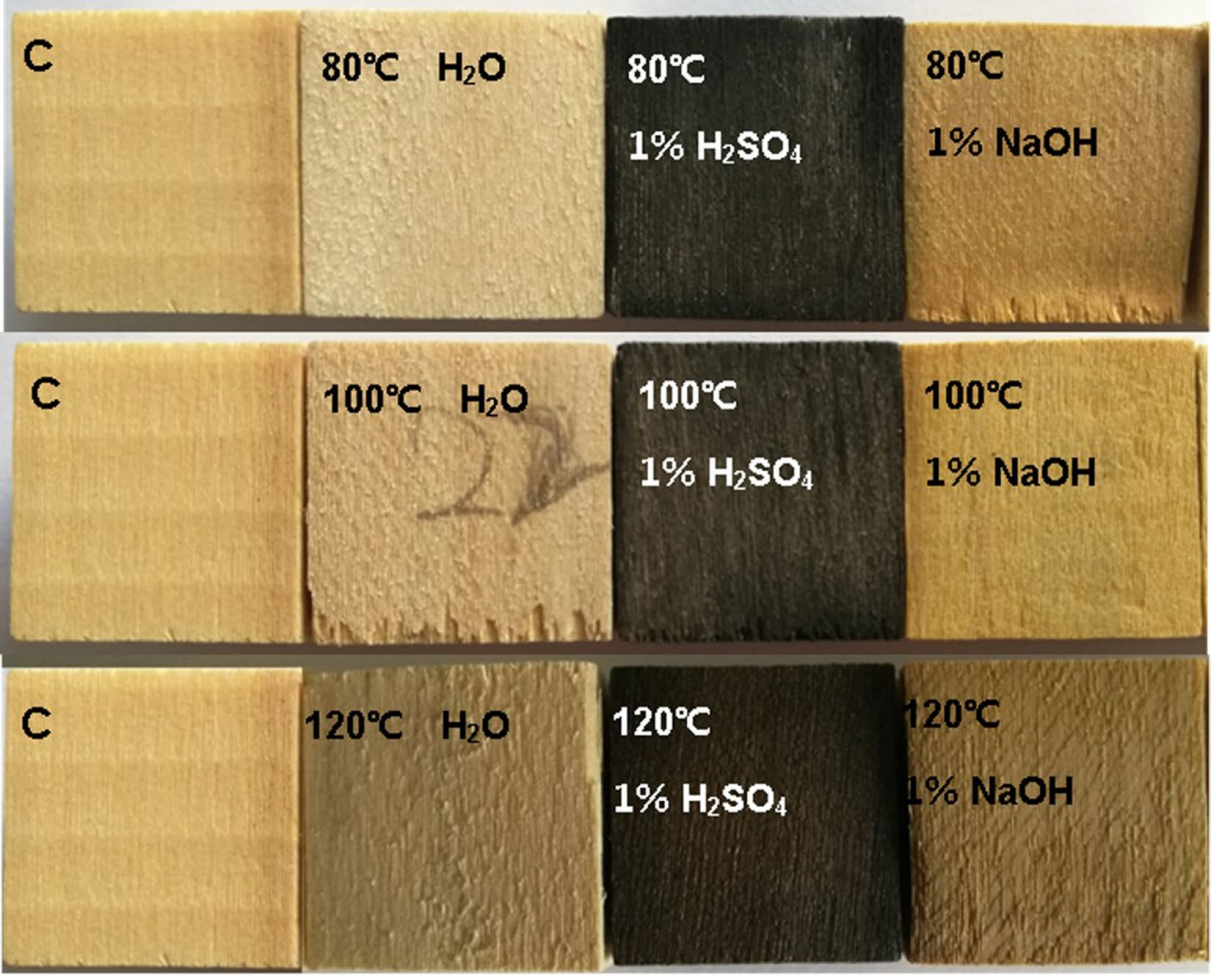
Changes in color after different treatment. C: untreated wood.

The poplar wood color changes on the tangential section after the thermal treatment was examined. Figure 1 shows the digital photographs of wood samples treated with hydrothermal and thermal treatments with H_2_SO_4_ and NaOH aqueous at various temperatures. The photos show the markedly changes and differences in different treatment conditions. The color of wood treated by hydrothermal treatment was light yellow like the pristine wood. Using the thermal treatment with H_2_SO_4_ aqueous, the wood color turned into dark chocolate-brown because of the acid degradation on wood tissues. As treatment temperature increased, no obvious changes were found. The wood thermal-treated with NaOH aqueous presented beige and easy to be distinguished from the untreated wood.

To quantify the color changes, the L* a* b* chromatic values were measured by the chromameter CR-5. Herein, L* is the lightness from 0 (dark) to 100 (white), a* is the red-green factor and b* is the yellow-blue factor, which is depending on whether there is a positive or negative value on the corresponding axis^11,12^. In other words, the increase of a* value means the color trends to red and the increase of b* value means the color trends to yellow. Table 1 shows that the L* values of all as-treated wood samples decreased compared to the pristine wood. Compared to other two treatments, the hydrothermal treatment had the minimum reduce degree. The L* value of acid aqueous thermal treated wood is only 33% than untreated wood and the temperature is little effect on its changes. The L* value decreased with the increase in temperature on the wood treated by thermal treatment with alkali aqueous. The lower L* value in treated wood indicates that the lower wood luminosity and higher darkening. The decrease of luminance (L*) with the increasing temperature during the hydrothermal treatment in this work mainly caused by the migrating or diffusing of extractives on wood surface^21^. The dehydration carbonizing of carbohydrate under sulfuric acid-based solution can be reasoned for the wood darkening in the 1% H_2_SO_4_ thermal treatment^22^. The decrease of luminance (L*) in thermal with 1% NaOH treatment wood should be caused by the partial hydrolysis of hemicellulose and oxidation of extractives^21,23^, and also related to the formation of new chromophore structures. The values of a* and b* dramatically decreased after the thermal treatment with acid aqueous and this implied that wood lost its colors. According to the former studies, the dehydration of carbohydrates in wood were responsible for the discoloration during the H_2_SO_4_ aqueous condition treatment. On the contrary, a* and b* increased with the increase in temperature after the thermal treatment with alkali aqueous. The increase of a* showed a progressive increase of red chromophores on the wood surface, and the increase of b* indicated that the thermal alkali treatment made the wood yellowing. Based on the previous reports, the removal of extractives, hydrolysis of hemicellulose, and the oxidation of these compounds on wood surface affected the color during hydrothermal and the thermal NaOH treatment resulted in color changes of the wood^21,23,24^. To verify the overall color changes of wood samples, △*E* was calculated by the follow equation^11^:

$${\rm{\Delta }}E={({\rm{\Delta }}{L}^{\ast 2}+{\rm{\Delta }}{a}^{\ast 2}+{\rm{\Delta }}{b}^{\ast 2})}^{1/2}.$$

The △*E* was increased up to 10.76 at 120 °C with the thermal water treatment. The greater color changes resulted from the acid and alkali treatments were due to the greater contribution from the chromaticity coordinates, namely, ΔL*, Δa* and Δb*. The Δ*E* variation is accordance with that in L* and suggested that the wood color was obviously changed in the thermal treatments with acid and alkali aqueous. Based on Cividini *et al*.^25^’s rule of color change distribution, the overall color after treatment were visible with naked eye. As mentioned previously, the total color difference △*E* was resulted from the cleaving of chemical bonds in hemicellulose or lignin, the formation of new chromophore substances and the movement of extractives.

**Table 1:** Changes in chromatic value and Standard Deviations after different treatment. C: untreated wood. (n = 3).

	C	Hydrothermally			Thermal with 1% H_2_SO_4_			Thermal with 1% NaOH		
		80 °C	100 °C	120 °C	80 °C	100 °C	120 °C	80 °C	100 °C	120 °C
L*	79.83 ± 1.51	79.44 ± 0.38	75.28 ± 4.76	70.50 ± 5.74	27.01 ± 4.35	37.14 ± 2.03	29.78 ± 6.06	66.54 ± 0.68	62.89 ± 1.15	54.50 ± 3.87
a*	4.87 ± 1.52	3.77 ± 0.02	4.66 ± 1.23	5.90 ± 1.81	−0.3 ± 0.16	0.28 ± 0.30	1.32 ± 1.03	4.73 ± 1.11	5.64 ± 0.77	7.49 ± 1.08
b*	24.75 ± 2.47	18.50 ± 0.19	19.33 ± 0.20	19.37 ± 0.52	2.05 ± 0.82	5.06 ± 0.58	5.16 ± 3.05	30.64 ± 0.16	26.53 ± 1.02	25.88 ± 0.94
*ΔE*		6.34	7.03	10.76	57.7	47.2	53.8	43.3	17.1	25.4

### Microstructure of wood cell wall changes

#### Hydrothermal treatment

**Figure 2:**
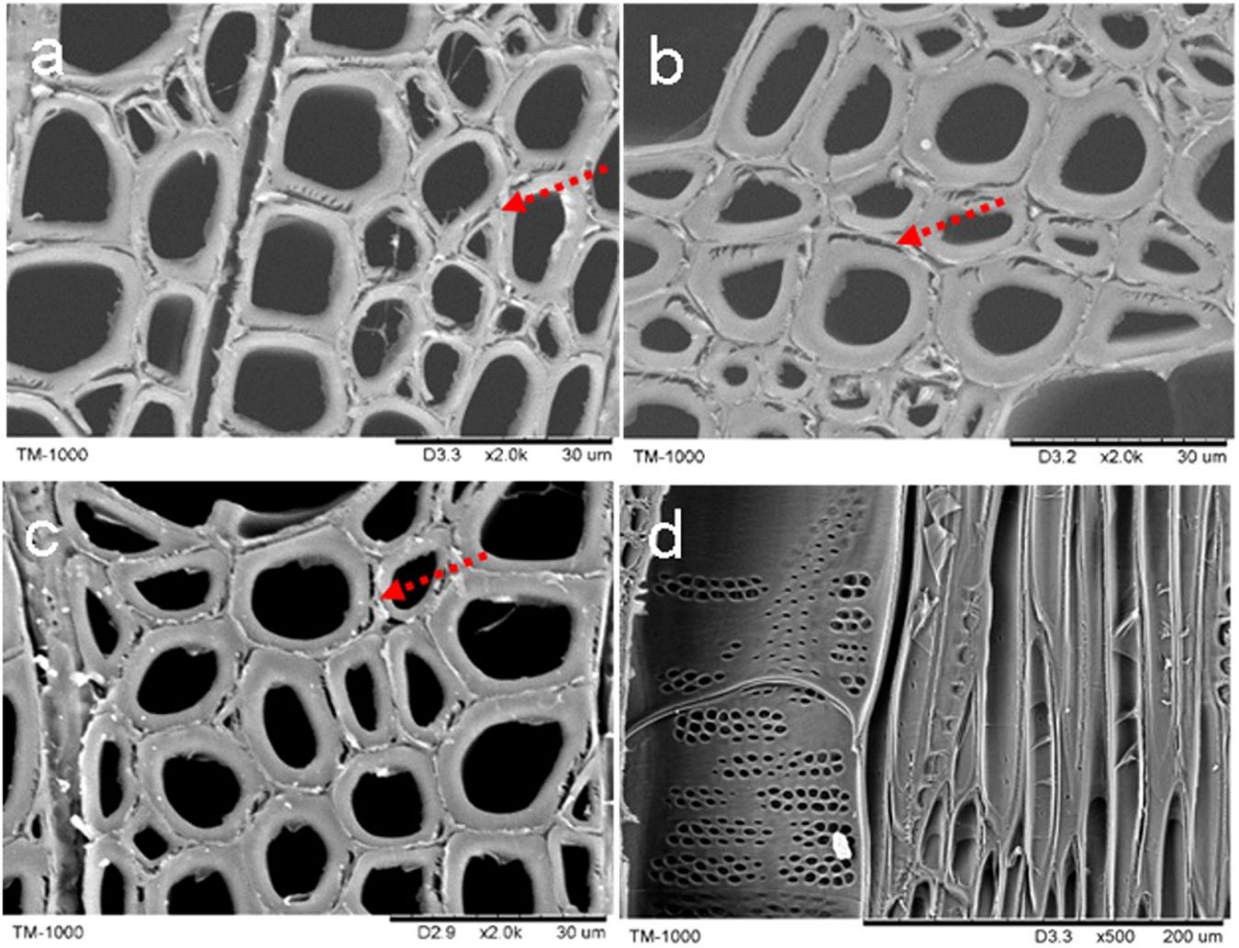
Wood cross section of hydrothermal treatment on 80 °C (**a**), 100 °C (**b**) and 120 °C (**c**), bar: 30 μm; radial section on 100 °C (**d**), bar: 200 μm. Red arrows show cracks on compound middle lamella.

It was observed that, on the cross section, the compound middle lamellas of wood cell were damaged after the hydrothermal treatment. The destruction was intensified with the increase in treatment temperature (Fig. 2a–c). There were some fractures on inter two fiber cells and direct along with the compound middle lamella at 80 °C hydrothermal (Fig. 2a), which were also observed at the 100 °C and 120 °C treatments (Fig. 2b,c). The radial section showed delamination between two fiber cells (Fig. 2d). Saito *et al*.^2^ reported the similar delamination phenomenon in adjacent fiber cells of *Eucalyptus globulus* wood, which was treated with the hydrothermal treatment for 10 minutes at 200 °C. According to the former hypothesis, the cell wall changes during the hydrothermal treatment would create thermal expansion stresses on wood. Generally, the free water content variation in wood will not alter cell wall structure when the water content is greater than the fiber saturation point. However, the hydrothermal treatment in an autoclave is a confined and limited space, so that the steam pressure and muggy field synergistic influence on the wood cell walls. With the increase in temperature, some amorphous polymers, such as lignin and hemicellulose, will be degraded. As well known, such polymers would show relatively high concentration in compound middle lamella^26,27^. Meanwhile, it was a binding site in two wood cells. Therefore, the stress concentration destroyed the cell walls, resulting in irreversible fractures. However, there were no fractures or destroyed on inter-vessel pits or fiber cell wall pits, which could be considered as the major water transport channels (Fig. 2d). To our knowledge, it is undeniable that some fractures in the cell wall due to all wood drying process, include the samples preparation^28,29^. But for SEM samples preparation, comparatively little distortion or damage where there is obvious damage to the pit membranes or other delicate structures^28^. And vacuum dried samples at 40 °C-60 °C is commonly used in many studies^1,3,5,29^.

#### Thermal with H_2_SO_4_ treatment

**Figure 3:**
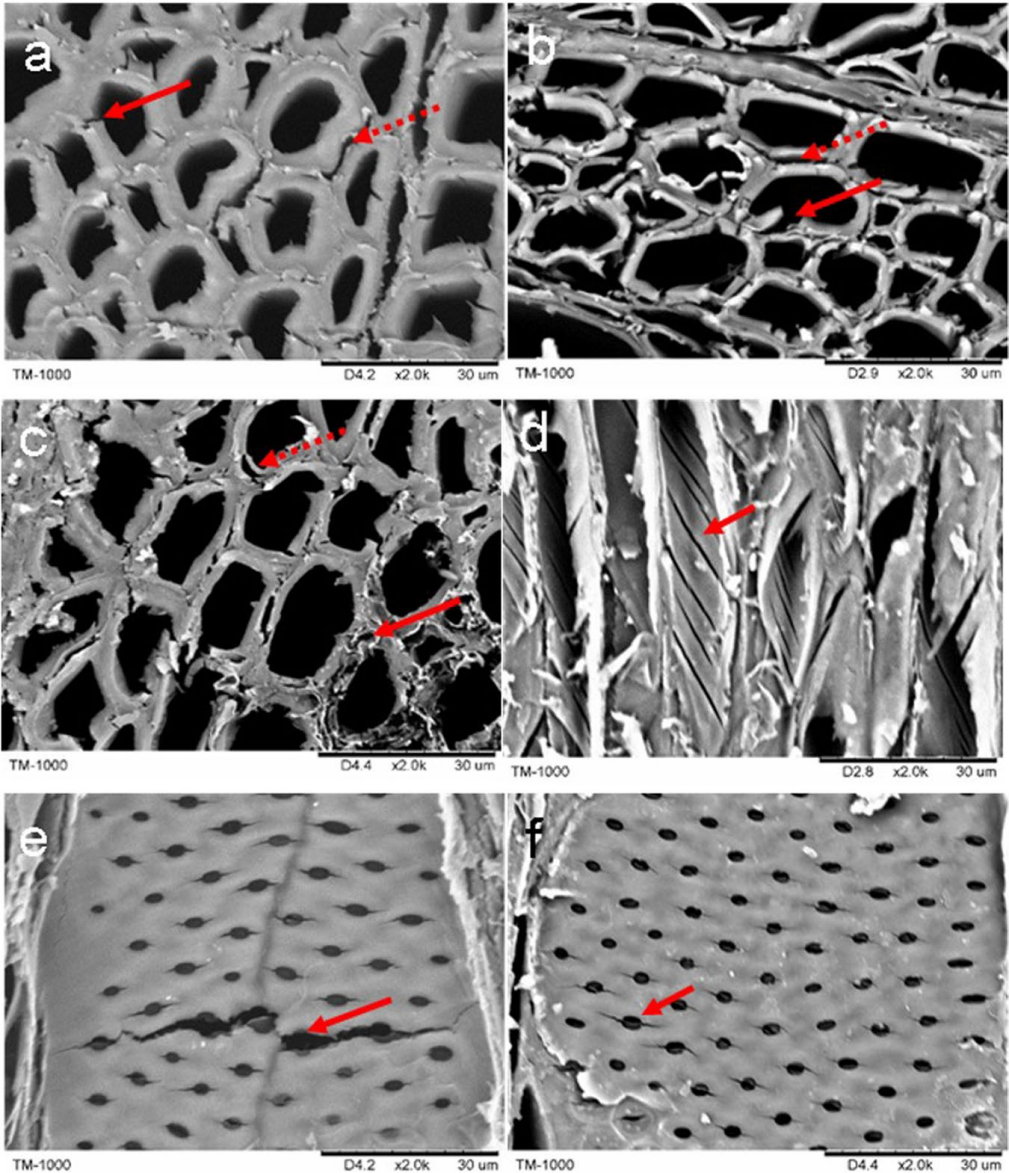
Cross sections after thermal with 1% H_2_SO_4_ treatment on 80 °C (**a**), 100 °C (**b**) and 120 °C, solid arrows and dotted arrows show cracks on cell wall and on compound middle lamella, respectively. Radial section on 120 °C after 1% H_2_SO_4_ treatment, solid arrows show cracks on fiber cell wall (**d**) and vessel wall pits (**e**,**f**) Bar: 30 μm.

After the thermal treatment with H_2_SO_4_ aqueous, the wood cell walls were severely broken on the cross section. These damages not only show fractures and peels in inter-cells but also existing numbers of deepen fractures on secondary cell wall (Fig. 3a–c). And these damages become seriously with increase of treatment temperature. In Fig. 3c, it was observed that the cell wall collapsed at 120 °C with the acid aqueous treatment. Most interesting, there were numerous fractures, which ran approximately parallel with S2 layer microfibril angle of wood fiber cell (Fig. 3d). Wardrop^30^ was observed a splintering failure that usually followed the microfibril angle of S2 layer and Senfat and Bendsten^31^ also found cell wall microchecks parallel to the microfibrils after repeated drying cycles. These phenomena are in accordance with our results. In addition, fractures also happened on pits of vessel wall after the 120 °C treatment, but not obviously at other temperatures (Fig. 3e,f). Vessel cell wall is easier collapse during wood thermal treatment in hardwood species, such as Birch, Poplar^32^. From the perspective of wood cell wall structure and composition, these fractures and damages can be explained as follows: (1) the H_2_SO_4_ thermal treatment degraded not only the soluble carbohydrates in lamella, but also on the secondary wall polysaccharides and lignin. The removal of hemicellulose and lignin from biomass was reported by many studies^33–35^. (2) The destruction of the intercellular layer was along with the cell gap expansion. In addition, the fractures orientation on cell secondary wall was closely related to MFA. In the secondary wall S2 layer, MFA is 10 °–30 ° to the tree growth direction, and hemicellulose and lignin is located on the outer surface of cellulose microfibrils as well as inter-fibrillar space^27^. The low degree of polymerization of hemicellulose made it easier degradation and produced fractures in the autoclave under pressure^36,37^. (3) The direction of fractures on vessel was also related to the microfibril arrangement. Generally, the vessel cell has a thin wall and disorder microfibril arrangement with MFA about 70°–90° to the wood longitudinal direction^37^. Wang *et al*.^38^ also found that some fibers were detached from each other and more pits opened cell lumina after diluting sulfuric acid treated aspen and birch wood. Same phenomenon was observed by Saito *et al*.^7^, the fiber tended to detach from each other after H_2_SO_4_ and hot water pretreatment, and the fibers were slightly or entirely separated from adjacent one. However, Pignali *et al*.^39^ showed that the dilute acid pretreatment altered poplar cell wall morphology from diffuse to a sharp and smooth interface. In fact, from our previous results, the wood cell wall would be rough and disintegration. Saito *et al*.^7^ found that that small spores were formed in the warty layer of innermost surface after the H_2_SO_4_ thermal treatment. But they also observed fractures on secondary wall after a hot water pretreatment.

#### Thermal with NaOH treatment

**Figure 4:**
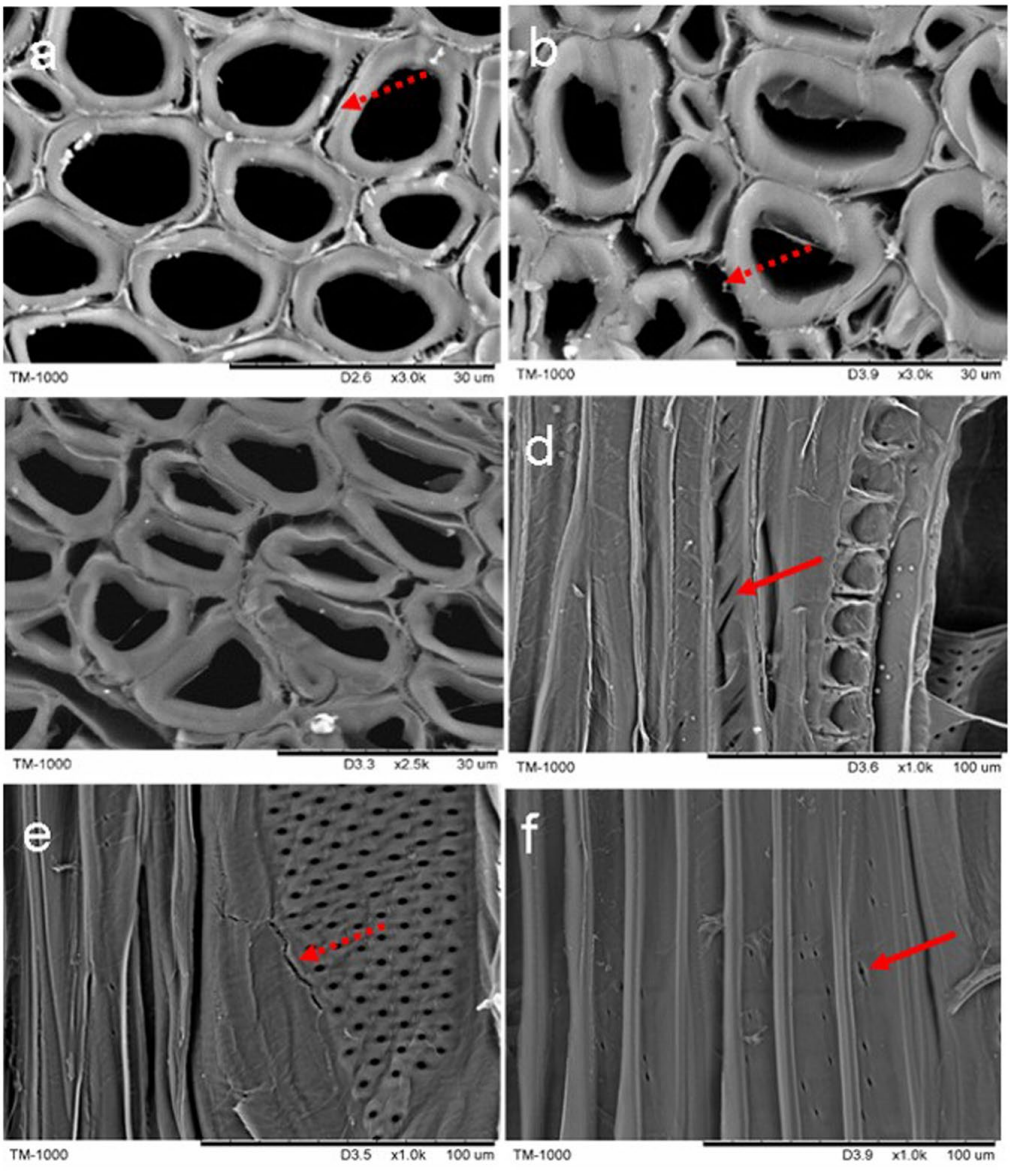
Cross sections after thermal with 1% NaOH treatment on 80 °C (**a**), 100 °C (**b**) and 120 °C, solid arrows and dotted arrows show cracks on cell wall and on compound middle lamella, respectively. Bar: 30 μm; Radial section on 120 °C after 1% NaOH treatment, solid arrows show cracks on fiber cell wall (**d**), vessel cell pits (**e**) and fiber wall pits (**f**) Bar: 100 μm.

Figure 4 shows the cross section of the wood after the NaOH thermal treatment. Although numerous fractures emerged in compound middle lamella and widened with increased temperature, the secondary cell wall of fiber was unbroken. In contrast, the cell wall was swelled and draped on the inter surface of cell lumen (Fig. 4a–c). On the radial section, the fiber cell wall emerged much more fractures at 100 °C and 120 °C with NaOH treatment (Fig. 4d,e). It was found that the fiber cells were separated from each other after the alkali treatment. The similar results were reported by Salehian *et al*.^40^. These suggested that the low temperature treatment made cell wall swelling, while the high temperature treatment caused hemicellulose degradation in thermal NaOH condition. Based on these findings, it was implied that the pore area in wood was increased in the NaOH thermal treatment, which was consistent with the previous report^41^. Generally, the alkaline treatment of biomass is effective in breaking the ester bonds among lignin, hemicellulose and cellulose^42^. As a result, the porosity of biomass could be increased after the removal or modification of lignin^40^.

As well known, alkali can easily penetrate the swollen cellulosic fibers and causes an increase in specific surfaces, making cell wall more accessible for chemical interaction. After thermal alkali treatment, the lignin was removed largely, the mechanical properties of cell wall decreased, the cellulose microfibril collapsed, and the porosity of the wood surface increased^8,37^^,^^41^. Satio K *et al*.^7^ reported that the warty layer was almost completely decomposed after the thermal 0.5% NaOH pretreatment. The microfibril dimeter of the *Eucalyptus* wood fiber was increased after the alkali treatments with different concentrations^43^. Similar to the NaOH treatment of wood, Belmokhtar^44^ reported the swollen cell wall and dissolved hemicellulose and lignin in liquid ammonia. The intercellular layer consists of low polymerization degree of polysaccharide, which is facile hydrolysis and peeling reaction of hemicellulose in the dilute alkali solution. Although the cell wall did not show significant damage, some fractures were observed on the fibers (Fig. 4d) and the vessel cell walls (Fig. 4e).

### Chemical structure analysis

**Figure 5:**
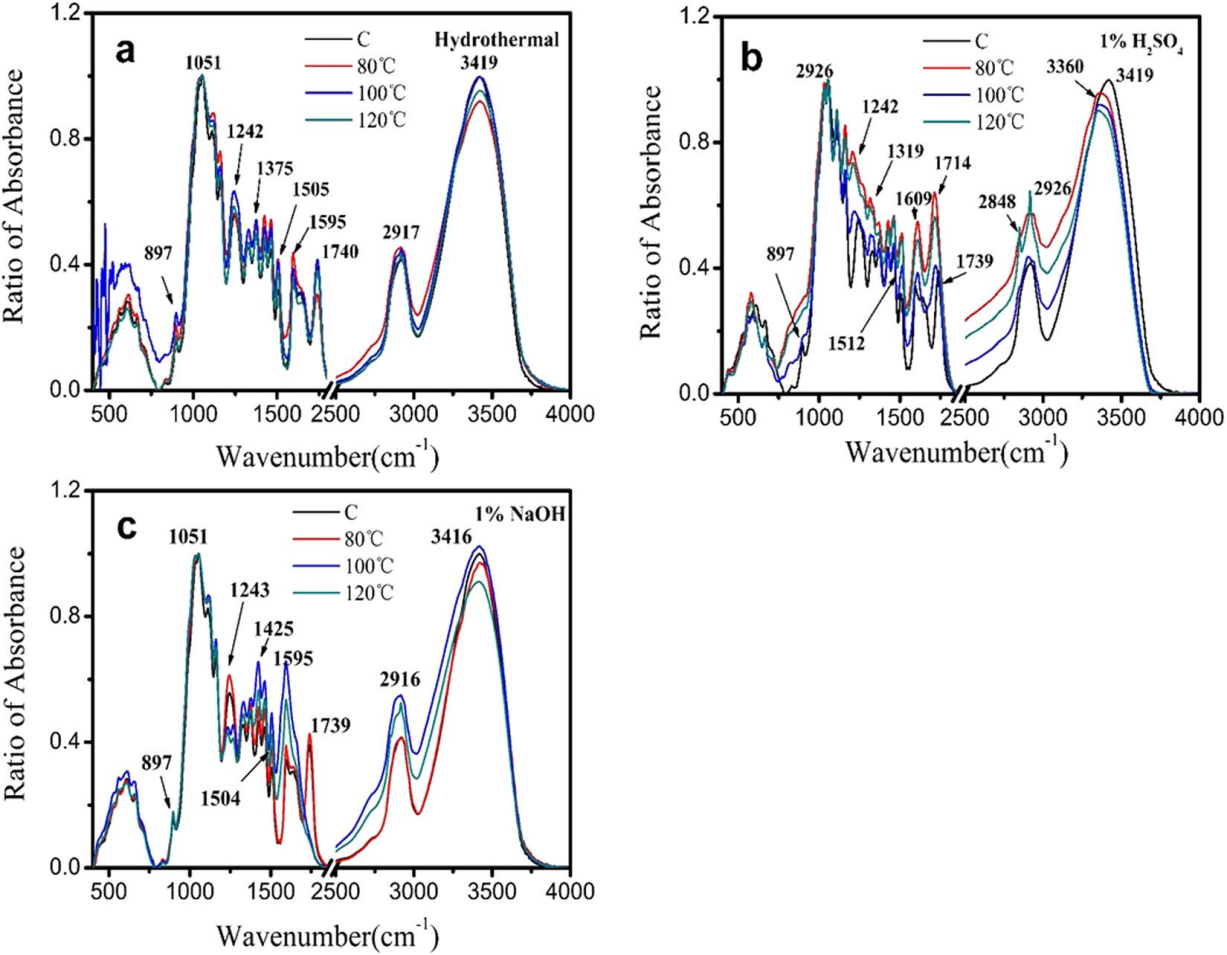
FT-IR spectra of hydrothermal (**a**), thermal with 1% H_2_SO_4_ (**b**), and thermal with 1% NaOH (**c**). Specific spectra were normalized at the maximum absorption band at nearly 1055 cm^−1^ as the internal standard to better compare differences in noted peaks. C is untreated wood samples.

Figure 5 shows the FTIR spectra of poplar wood blocks treated by thermal conditions of water, H_2_SO_4_ and NaOH aqueous. Compared to the untreated wood, similar bands can be observed in thermal water treated samples. However, the band positions and intensity were dramatically changed by the reaction of acid and alkali with wood compounds. For example, the bands at 1739 cm^−1^ to unconjugated C=O stretching vibration, 1595 cm^−1^ to aromatic skeletal vibration in lignin, 897 cm^−1^ to C−H out of phase ring stretching in cellulose vanished in thermal 1% H_2_SO_4_ treated samples. The band at 1504 cm^−1^ to aromatic ring vibrations shifted to 1512 cm^−1^. It was also worth noting that the band at 1246 cm^−1^ to C−O-C stretching for syringyl ring and xylan also weakened. All the mentioned bands were attributed to the previous studies^45,46^. Lehto *et al*.^47^ reported that, with an increase in pretreatment severity, birch wood chips caused a clear decrease of relative intensity in ester structures at the wavenumber of 1730 cm^−1^ and caused significant removal of hemicellulose from wood materials. All of these implied that lignin, hemicellulose and partly cellulose can undergo various degree degradation during the thermal acid aqueous treatment. Meanwhile, some new chemical bonds and groups formed in this harsh treatment. As shown in Fig. 5B, some new bands appeared in the acid treated wood, such as the band at 1714 cm^−1^ being typical of the C=O stretching in unconjugated ketones, and the band at 1609 cm^−1^ attributed to C=C stretching vibrations of the aromatic ring (lignin). This could be due to the cleavage of mainly β-O-4 linkages in lignin with carbohydrates and formation of newly condensed lignin structures^48^^,^^49^. Sannigrahi *et al*.^50^ previously reported the band at 1714 cm^−1^ was associated with the formation of pseudo-lignin from holocellulose. The clear band at 1609 cm^−1^ and disappearance of 1595 cm^−1^ were attributed to the two types of lignin monomer, i.e., syringyl and guaiacyl nuclei, respectively. It was indicated that the lignin was partly degraded in acid, but guaiacyl nuclei was degraded before syringyl nuclei. In other words, syringyl lignin was more stable during the acid treatment compared to guaiacyl lignin. Guaiacyl (G units) was enhanced degradation with the increased pretreatment temperatures or addition of acidic solution under thermal chemical treatment^51^. The band positions and intensity of wood in the thermal 1% NaOH treatment at 80 °C showed no difference with the untreated wood. However, with the temperature increased, the bands were significantly different. Such as the disappearance of band at 1740 cm^−1^, the obviously weakened band at 1243 cm^−1^. It was implied that the thermal 1% NaOH aqueous treatment mainly caused the deacetylation reaction of xylan, which meant the decomposition of hemicellulose. This consisted with previous findings, which showed that 28% xylan and 35% lignin could be removed by the 60 min NaOH pretreatment at 120 °C^41^. The changes of chemicals during the thermochemical treatment was also responsible for the changes in wood color and the breaking of wood cell walls.

## Conclusions

The effects of thermal treatments with water, H_2_SO_4_ and NaOH aqueous on the color, cell wall and the chemical structure of poplar wood were investigated. The changes of wood tangential surface color is connected with the thermal treatment solution type and temperature. The lightness value decreased in all thermal liquid treatments. The a* value increased with temperature in all liquid treatments. The b* value increased with temperature in hydrothermal and thermal with H_2_SO_4_ treatment but decreased with temperature in thermal with NaOH treatment. The total color difference (Δ*E*) was slightly changed in the hydrothermal treatment, but dramatically changed in the thermal with 1% H_2_SO_4_ and 1% NaOH aqueous treatments. Only middle lamella layers fractured in the thermal water treatments and notably with the increased treatment temperature. In the thermal H_2_SO_4_ treatment, owing to the acid degradation both middle lamella layers and secondary cell wall were destroyed severely, especially at a higher temperature. Several fractures were observed on the fiber cell walls and they usually parallel to the S2 layer microfibril angle (MFA). The pits on vessel wall damaged in the thermal H_2_SO_4_ treatment and severely in higher temperature treatment. Similar to the hydrothermal treatment, the middle lamella layers were mainly destroyed after the thermal NaOH aqueous treatment. The FTIR analysis suggested that the chemical structure had no obvious changes in the H_2_O treatment but was distinctively different in the H_2_SO_4_ and NaOH treatments, particularly being observed on the missing or decreasing C=O absorption peak, which was a characteristic peak of hemicellulose. Hemicellulose is degraded and new compounds produced during thermal with H_2_SO_4_ and NaOH treatment. This maybe can account for the color changes in such treatment. On the other hand, lignin was partly degraded in the H_2_SO_4_ treatment and guaiacyl nuclei was degraded before syringyl nuclei. The deacetylation reaction of xylan in hemicellulose was accelerated with the increased temperature in the NaOH treatment. The comparative study of different treatments using microscopic observations and chemical analysis led to a better understanding of changes on surface color and cell wall structure of the bulk wood after thermochemical treatments.

## Experimental

### Materials

The plantation Poplar wood was harvested from State-owned Jiaozuo forest farm, Henan Province, China. 10 cm thickness lumber disc from the breast high of tree was stored in laboratory. After being air-dried, the wood was cut to a standard size of 2 cm × 2 cm × 2 cm (Longitudinal × Radial × Tangential). The wood samples were washed by distilled water and oven dried at 50 °C. The final average moisture of wood samples was 4.6%.

### Thermal treatments with water, 1% H_2_SO_4_ and 1% NaOH aqueous

The concentration of H_2_SO_4_ and NaOH aqueous solution was mass ratio with distilled water. In a typical process, 80 mL different liquids were prepared in three beakers and stirring for a few minutes. The as-prepared wood blocks were added into the different liquids and mixed. Subsequently, the mixture was placed in autoclaves those were heated to 80 °C, 100 °C and 120 °C and maintained the temperatures for 2 hours, and then cooled to room temperature. Finally, the treated wood samples were cleaned by distilled water several times followed by vacuum drying at 60 °C. There were three replicates for each treatment condition.

### Test of chromaticity value

The color changes during the thermal water, 1% H_2_SO_4_ and 1% NaOH aqueous treatments of wood tangential section were measured by the chromameter CR-5 (Konica Minolta, INC, Japan) under optional target mask (3 mm diameter) and the D65 light source (Daylight, color temperature of 6504 K). The measurement type was Reflectance mode at 10° observer angle and specular component was excluded. Color measurements were taken on 6 locations on each sample and mean values were calculated for each wood sample. We marked six blocks with same area on the wood surfaces and superposed the light center with every block on the target mask. The color was described using the CIEL*a*b* system, which was established by International Commission on Illumination in 1976. The CIEL*a*b* system consists of three perpendicular axes, where L* axis is the lightness, a* axis is the ratio of red to green and b* axis is the ratio of yellow to blue^11,13^. All three replicates in each treatment condition were measured.

### SEM and FTIR

The as-treated wood was cut into one cm^3^ cube and the surface was carefully smoothened on a microtome. Then the sample was coated with gold sputtering for about 20 s. Finally, the cell wall microstructure was examined using the scanning electron microscopy (Hitachi TM-1000, Japan) at an accelerating voltage of 15 kV. The chemical structure of the treated wood samples was investigated by the FTIR spectrscopy. All samples were cut into small pieces and finely powder and then formed film with moderate KBr. The spectra were recorded using a FTIR spectrometer (Nicolet 360, Thermo Scientific, U.S.A) between 400 cm^−1^ and 4000 cm^−1^ with an average of 40 scans and a resolution of 4 cm^−1^. Before further compare analysis, all peaks were normalized at the maximum absorption band at nearly 1055 cm^−1^ as the internal standard^26,52^. For each sample, the values from the three spectra were accumulated and averaged. Peak heights of absorption bands were measured by the OMNIC software (Version 8.0, Nicolet Instruments Corporation, and U.S.A).
